# Assessment of the Baby Friendly Hospital Initiative Implementation in the Eastern Mediterranean Region

**DOI:** 10.3390/children5030041

**Published:** 2018-03-11

**Authors:** Ayoub Al-Jawaldeh, Azza Abul-Fadl

**Affiliations:** 1 Department of Nutrition Sciencies, University of Vienna, Viena A-1090, Austria; aljawaldeha@who.int; 2 Pediatric Department, Banha Faculty of Medicine, Benha University, Benha 13511, Egypt

**Keywords:** breastfeeding promotion, Baby Friendly Hospital Initiative, malnutrition, exclusive breastfeeding, obesity, Code of marketing of breastmilk substitutes

## Abstract

The Baby-Friendly Hospital Initiative (BFHI) is a global program for promoting support and protection for breastfeeding. However, its impact on malnutrition, especially in countries of the Eastern Mediterranean region (EMR) that are facing the turmoil of conflict and emergencies, deserves further investigation. Having said that, this paper aims to discuss the status and challenges to BFHI implementation in the EMR countries. Data on BFHI implementation, breastfeeding practices, and nutritional status were collected from countries through structured questionnaires, personal interviews, and databases. The 22 countries of the EMR were categorized as follows: 8 countries in advanced nutrition transition stage (group I), 5 countries in early nutrition transition stage (group II), 4 countries with significant undernutrition (group III), and 5 countries in complex emergency (group IV). The challenges to BFHI implementation were discussed in relation to malnutrition. BFHI was not implemented in 22.7% of EMR countries. Designated Baby-Friendly hospitals totaled 829 (group I: 78.4%, group II: 9.05%; group III: 7.36%; group: IV5.19%). Countries with advanced nutrition transition had the highest implementation of BFHI but the lowest breastfeeding continuity rates. On the other hand, poor nutritional status and emergency states were linked with low BFHI implementation and low exclusive breastfeeding rates but high continuity rates. Early initiation and longer duration of breastfeeding correlated negatively with overweight and obesity (*p* < 0.001). In countries with emergency states, breastfeeding continues to be the main source of nourishment. However, suboptimal breastfeeding practices prevail because of poor BFHI implementation which consequently leads to malnutrition. Political willpower and community-based initiatives are needed to promote breastfeeding and strengthen BFHI in the region.

## 1. Background

The Eastern Mediterranean region (EMR) is witnessing a rising burden of disease due to undernutrition, micronutrient deficiencies, overweight, and obesity as a consequence of both emergency situations and nutrition transition that are placing different communities in the region at various nutrition-related health risks [1]. The overall prevalence of malnutrition in the region remains well below the world average [2,3]. The economic and societal costs to developing countries who are failing to achieve a reduction in hunger and undernutrition, including micronutrient result in an annual loss of five million children and 220 million disability-adjusted life years. Childhood and maternal undernutrition result in loss of billions of dollars from reduced productivity and lost incomes in these countries. The developing countries have fallen far short of the pace required to meet their Sustainable Development Goals (SDGs) [4].

Exclusive breastfeeding for the first six months and continued breastfeeding for two years benefit both mothers and children by preventing many diseases in the short and long term, [5] as well as impacting the economy of nations by impacting the intelligence, educational attainment, and income later in life [6,7]. Breastfeeding patterns impact nutritional status and can reduce overweight and obesity rates [8], which are alarmingly high in the EMR ranging from 1.9% to 21.9% among preschool children (<5 years) [9,10] and are linked to the rising mortality in the region from noncommunicable diseases (NCDs) [11,12]. Available studies highlight low rates of exclusive breastfeeding (<6 months) in most countries of the region, with the regional average being of 31.8 in 2012. The lowest rates were documented in Somalia (5.3%) and Tunisia (8.1%). While the highest rate reported in Afghanistan (58%), in other countries, such as Egypt, Syria, Djibouti, Pakistan, Palestine, and Sudan, the rate is around 40% [13]. The region has to increase and sustain annual increases of 1.2 percentage points in the rate of exclusive breastfeeding between 2012–2025 in order to meet the World Health Organization (WHO) global nutrition target which is more than 50% exclusive breastfeeding. However, as per rates reported by United Nations International Children's Emergency Fund (UNICEF) for the Middle East in 2016, exclusive breastfeeding rates for infants at six months of age in the Eastern Mediterranean region have considerably declined [14,15].

According to UNICEF, improving breastfeeding practices worldwide could save the lives of an estimated 1.5 million children annually [16]. Furthermore, the risk of mortality before the age of five years is expected to decrease from 13% to 11.6% if optimal breastfeeding practices are implemented. The Baby-Friendly Hospital Initiative (BFHI) is one way to achieve this goal, especially in that it emphasizes Code implementation and ending free and low-cost supplies. However, the BFHI has been facing many challenges with implementation and designation of hospitals worldwide and particularly in the region over the past decade. A global report on national implementation [13] showed that out of a total of 117 countries, 86% reported that they had implemented the BFHI and 71% reported that they had a BFHI programme as of 2016–2017. However, only one in five had ever designated more than one-half of their maternity facilities as Baby-Friendly [13].

The aim of this paper is to review the status and progress of BFHI implementation in the EMR countries in relation to the nutritional status of children under five and to discuss challenges towards achieving universal BFHI.

## 2. Methods

Data were collected from each of the 22 countries of the region using a short survey that was distributed to focal persons in the government responsible for the implementation of the BFHI programme in each country under the EMR. The countries presented their data from recent national surveys or registries. The data for the breastfeeding rates and nutritional indices were obtained from the WHO regional data banks [14]. Additional data for BFHI status was obtained from UNICEF report from five countries; Morocco, Sudan, Djibouti, Oman, and Tunisia (UNICEF, 2016). The survey included questions about the number of currently designated and previously designated Baby-Friendly hospitals and constraints during implementation and assessment. The data was analyzed, and confirmation of the data collected was obtained by directly contacting country officials. Suggestions and recommendations for action were obtained by one-to-one interviews with the focal nutrition country representatives and presented as a qualitative analysis.

This study focused on the 22 countries of the EMR. These countries are included in the region based on the request of member states who decide which region they wish to become affiliated with. They present their request to the WHO regional committee and World Health Assembly (WHA), which decides the list of countries included within each region. Hence the Eastern Mediterranean region is represented by 22 countries that include Afghanistan, Bahrain, Djibouti, Egypt, Jordan, Iran, Iraq, Kuwait, Lebanon, Libya, Morocco, the occupied territory of Palestine, Oman, Pakistan, Qatar, Saudi Arabia, Somalia, Sudan, Syria, Tunisia, the United Arab Emirates, and Yemen (in alphabetical order). The focus of our study was related to the above-mentioned 22 countries only, irrespective of geographic or other considerations.

In this study, countries were grouped according to a classification made by the Regional Committee for the EM/RC57/4 Regional strategy on nutrition for the Eastern Mediterranean on September 2010 in its 57th session [3]. Accordingly, the countries were divided into four groups, or country clusters, with regard to nutrition stages and dominant nutrition problems. These four groups were categorized as countries in advanced nutrition transition stage, countries in early nutrition transition stage, countries with significant undernutrition, and countries in a complex emergency [3]. This categorization was used in the current analysis of the BFHI implementation to link BFHI implementation to the nutrition stages and dominant nutrition problems. The status of the BFHI program was assessed by the percent of Baby-Friendly hospitals designated in a country. The countries were grouped as follows:

Group I included eight countries: the Islamic Republic of Iran, Tunisia, and member countries of the Gulf Cooperation Council (GCC) including Bahrain, Kuwait, Oman, Qatar, Saudi Arabia, and the United Arab Emirates (UAE).

Group II included five countries: Egypt, Jordan, Lebanon, Morocco, and Palestine.

Group III included four countries that were reported to have significant undernutrition particularly high levels of acute and chronic child malnutrition, widespread micronutrient deficiencies, and emerging overweight, obesity, and malnutrition of affluence in certain socioeconomic subgroups. These included Djibouti, Iraq, Sudan, and Pakistan.

Group IV included five countries with complex emergency situations with severe child and maternal undernutrition and widespread micronutrient deficiencies. They included Afghanistan, Libya, Somalia, Syria, and Yemen.

The countries were compared in relation to the number and percent of Baby-Friendly hospitals designated over the past decade. Trends in BFHI were compared in the above groups.

Breastfeeding practices were assessed by rates for timely initiation of breastfeeding in the first hour, exclusive breastfeeding (EBF) for six months and continued breastfeeding at 12 and 24 months (expressed in percent). Nutritional status was assessed by rates for underweight, stunting, overweight, and obesity (expressed in percent).

The BFHI indicators were correlated with malnutrition including the status of underweight, stunting, overweight and obesity.

The impediments and barriers to implementation and sustaining of the BFHI program were assessed by feedback from the focal nutrition officers from the 22 countries of the region through survey forms and one-to-one interviews. The feedback was qualitatively analyzed and presented in the discussion.

### 2.1. Statistical Analysis

We used frequency distribution presented as percentages and means that were presented in tables using Excel 2010. Student *t*-test (for parametric data) and Mann-Whitney *U* test (for non-parametric data) were used for comparison of two distinct groups. For the comparison, more than two groups, analysis of variance with post hoc Bonferroni test (for parametric data) and Kruskal-Wallis test followed by Mann-Whitney *U* test (for non-parametric data) were used. Categorical variables were compared using Chi-square and Fisher exact tests. Spearman rank correlation coefficient (*r*) was used to measure the strength and direction of a linear relationship between two variables. The level of significance cut off used was *p* < 0.05.

## 3. Results

Table 1 presents the status of Baby-Friendly hospitals categorized by country groups in various nutrition stages and with dominant nutrition problems. Group I presented the eight countries in advanced nutrition transition and included Iran and Tunisia and all member countries of the GCC except Yemen. The number of Baby-Friendly hospitals was 650 constituting around 80% (78.12%) of hospitals that were designated in the region. Group II presents the five countries in early nutrition transition in which the number of reported designated Baby-Friendly hospitals was 57, constituting 6.85% of the designated hospitals in the region. Group III presents the four countries that were reported to have significant undernutrition and included 86 designated Baby-Friendly hospitals, constituting 10.34% of the designated hospitals in the region. Group IV presents the five countries in a complex emergency with severe child and maternal undernutrition and widespread micronutrient deficiencies. The number of designated hospitals in this group of countries was 39 representing 4.69% of the designated hospitals in the region.

**Figure 1:**
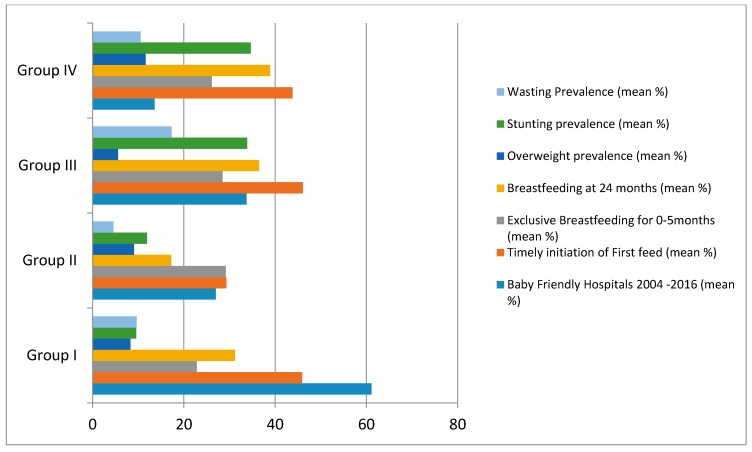
Bar chart illustrating the relationships between nutritional status in the different groups under study and breastfeeding and Baby-Friendly status in the respective group (mean % designates the mean percentage prevalence for each country by group in the region under study).

Table 2 and Figure 1 present the trends in Baby-Friendly hospitals over the past 12 years (2004 to 2016). The table compares the percent Baby-Friendly hospitals over this period. It shows that in group I countries characterized by advanced nutrition transition, most of the countries achieved high Baby-Friendly status and were able to maintain their achievements over time. In group II countries with early nutrition transition, the countries had high implementation rates at the beginning of the period but then the percent hospitals maintaining Baby-Friendly status dropped. In group III countries with acute and chronic malnutrition, the implementation rates were low or absent in three out of the 4 countries in the group. In Pakistan, it was low but then picked up at the end of the period, while in Iraq, the trend was in the opposite direction, i.e., it was high at the beginning of the period then dropped to one third by the end of the period. In group IV countries with complex emergency situations, implementation rates were very low at the beginning of the period in all three countries and remained low but seemed to show a steep rise at the very end of the period only for Afghanistan from 1% to 17% and not for Sudan or Somalia.

**Figure 2:**
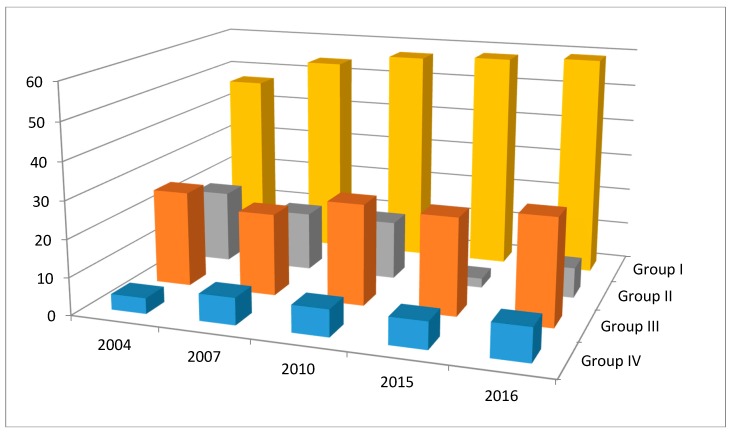
Comparison of trends in percent of Baby-Friendly hospitals designated from the period 2004 to 2016 in the four groups of the 22 countries of the Eastern Mediterranean region of the World Health Organization. Country Groups include 8 countries in advanced nutrition transition stage (group I), 5 countries in early nutrition transition stage (group II), 4 countries with significant undernutrition (group III), and 5 countries in complex emergency (group IV).

Table 3 and Figure 2 compare the BFHI, breastfeeding indicators, and nutritional indices in groups under study. The table and figure demonstrate significant differences in nutritional status between groups with the rising trends in overweight and obesity in the countries with advanced nutrition transition and high rates of stunting and wasting in the other groups of countries especially those in complex emergency situations. Exclusive breastfeeding was consistently low in all groups despite the tendency increase of overweight in groups I and II and stunting and wasting to increase in groups III and IV. Correlation studies between breastfeeding and nutritional status for all the countries were significant for continued breastfeeding at 12 months with stunting and wasting (*r* = 0.69, *r* = 0.67 respectively) at *p* < 0.01 and 24 months (*r* = 0.9 and *r* = 0.87 respectively) at *p* < 0.001. Timely first suckle correlated highly with overweight (*r* = 0.5) at *p* < 0.05 and obesity (*r* = 0.5) at *p* < 0.05. Obesity correlated significantly with continued breastfeeding at 12 months (*r* = 0.48) at *p* < 0.01. Overweight and obesity correlated poorly with the continued breastfeeding rates at 24 months (*r* = 0.36, *r* = 0.33) and respectively at *p* > 0.05 as shown in Table 3.

## 4. Discussion

Overall progress in BFHI implementation is high in countries with advanced nutrition transition. These countries have high levels of overweight and obesity and moderate levels of undernutrition and micronutrient deficiencies in some population subgroups. They include Iran, Tunisia, and all member countries of the GCC except Yemen. These countries have high implementation and designation rates of Baby-Friendly hospitals and are able to maintain their gains over time. This is probably because the GCC countries are driven by the economy and political willpower that keep them closely abridged with European and Western communities. However, these GCC countries have suboptimal breastfeeding practices [16] particularly among nationals and the poorly educated immigrants from very poor countries [19,20,21]. Despite Islamic teachings that support continued breastfeeding for two years and evidence suggesting that there are many acute and chronic diseases linked with not breastfeeding for two years [16], suboptimal breastfeeding leading to early discontinuation of breastfeeding is still commonly practiced in the GCC countries.

Countries characterized by early nutrition transition have low and declining BFHI implementation impeding the BFHI designation process. These countries are characterized by moderate levels of overweight and obesity. Moderate levels of undernutrition prevail in specific population groups especially women and children. Also, there are significant differences in social class leading to widespread regional disparities between urban and rural areas. Micronutrient deficiencies, especially iron deficiency, continue to prevail [1]. Iran, Egypt, Jordan, Lebanon, Libya, Morocco, Palestine, and Syria are included in this group. Lack of funding sources, engagement in political instability, and country priorities focused on emergency situations make them unable to recognize the short and long-term benefits of BFHI and its impact on their economy. These countries are looking for quick wins, harvesting of cash, and making quick profits to stabilize their governments and overcome increasing poverty. Hence, these countries are engaged in providing medical supplies and even of supplies of infant milk formula rather than investing in prevention by making their healthcare facilities become Baby-Friendly. Countries with acute and chronic malnutrition, as well as countries with complex emergency situations, have poorly implemented BFHI programs and no designated maternity facilities. BFHI can be beneficial to these countries and should be included in the training packages directed to healthcare staff working under emergency situations [22].

The rising trends in overweight status, which are responsible for the increasing burden of NCDs in the region, were observed to be directly negatively correlated with the duration of breastfeeding—the shorter the duration of breastfeeding, the higher the prevalence of overweight [23]. The double burden of undernutrition and overnutrition in low- and middle-income countries potentially increases their risk to both communicable diseases and NCDs and thereby increasing mortality rates [24]. This is an impediment to progress toward achieving their SDGs [25]. Hence, promoting and supporting breastfeeding; particularly continued breastfeeding for two years, which can directly impact the rising trends in overweight status, decrease the increasing burden of NCDs in the region, and assist countries in achieving their SDGs [25,26].

In this study, there were many lessons learned, as reported from the focal nutrition country coordinators of the EMR. The implementation process of BFHI faces many challenges that impede and delay the designation process and lead to the low exclusive breastfeeding rates in the region. Examples from Saudi Arabia show how a country can face the increased pressure from industry by increasing its designated Baby-Friendly hospitals to promote breastfeeding [18]. According to Victora et al. [7] the scaling up of breastfeeding to a near universal level could prevent 823,000 annual deaths in children younger than five years and prevent 20,000 annual deaths from breast cancer. Hence, promotion of breastfeeding in the EMR countries can benefit both developed and developing countries.

Feedback from countries report that expansion through capacity building of health staff in the hospitals preparing to become Baby-Friendly was seen to work in many countries as Egypt, Morocco, Kuwait, Iraq, Syria, and Saudi Arabia. However, this was met by a rapid turnover especially of the top management and junior staff who were responsible for implementation. Institutionalization of BFHI as a national program, as shown in Egypt and Morocco, impacted progress in BFHI. This was supported by legislative acts and ministerial decrees for urging hospitals to become Baby-Friendly and abide by the Code, as in the case of Egypt [27]. In other countries such as Syria and Saudi Arabia, strengthening of the regulatory, monitoring system, and feedback mechanisms for BFHI activities was effective in achieving and sustaining BFHI.

Feedback from countries interviewed showed that advocacy was a buy-in for government sectors and that getting political commitment and involvement was important for the success of BFHI. Having legislative and regulatory circulars to pressure the facilities into becoming Baby-Friendly was useful for accelerating BFHI implementation in Egypt, Morocco, and Afghanistan. Egypt was a case study for updating training guidelines/educational materials for faculty staff for teaching graduates the principles of BFHI. In Egypt, encouraging research related to BFHI was used to obtain baselines and innovative approaches for BFHI implementation. Multi-sectorial approach and involvement of multiple stakeholders in BFHI implementation were useful in blending BFHI into the community as in the case of Kuwait, Egypt. These findings are supported by similar findings from the national report on the global implementation survey for BFHI released in 2017 by the WHO team in Geneva [13].

The designation of the private hospitals as Baby-Friendly is a case study for BFHI in Kuwait. In Kuwait and Saudi Arabia, BFHI extended beyond the hospital to the community to make primary health centers Baby-Friendly. The UAE was the only country to designate the Al-Sharjah Emirate as Baby-Friendly. The key to the UAE’s success is both political commitment and support from their First Lady from the royal family, as well as the success of their mother-to-mother support group initiative. Political commitment supported BFHI in Kuwait and Lebanon. In Saudi Arabia, BFHI educational materials and applications on smartphones, Twitter, and Instagram accounts are useful for reaching out to mothers and the general public. Other workers have shown that social media is instrumental in the promotion of healthcare messages [28].

The main common impediments to implementation in most countries included deficient knowledge of health professionals in BFHI and their role in supporting mothers in overcoming breastfeeding-related difficulties. Another important and common-place challenge is the continued manipulation by industry and their influence on country policies and media to market and distribute their products freely to health workers and mothers. Despite the various forms of crises that many of the countries are facing in the region, there is no emergency preparedness plan to ensure an integrated response to protect and support BFHI programs as an important measure for ensuring the survival of mothers and babies living in countries with emergencies. The reversal of BFHI trends in countries of the region is due to the lack of sufficient political buy-in and increasing instability in many countries in addition to the high staff turnover in hospitals that leads to the lack of retention of the trained staff and committed administrators. Studies have shown that the integration of breastfeeding management skills in the curriculum for residents in pediatrics, family medicine, and obstetrics and gynecology improves services and increases breastfeeding support [29]. Hence, the importance of integrating BFHI practices in the medical curricula of medical and nursing schools and the use of political buy-in as a tool for going to scale at the national level for ensuring sustainability.

## 5. Conclusions

We conclude that BFHI implementation impacts nutritional status in the EMR countries, and this is linked to the current status of instability. Protection and support of breastfeeding are recommended in order to assist countries in conflict to face the challenges of emergency situations. Breastfeeding promotion through BFHI can benefit both affluent and poor countries [7]. All governments need to integrate BFHI into their national action plans for strengthening their health delivery services and preparedness for emergencies. Political enforcement of BFHI implementation can assist in expanding the designation process of Baby-Friendly hospitals. Strengthening monitoring and quality management of the BFHI programme can ensure sustainability through a periodic reassessment of hospitals. Updating and strengthening of undergraduate, pre-service, and in-service breastfeeding education and training ensure change in practices over the long term.

An important strategy that requires political commitment is to protect breastfeeding by strengthening control over free distribution and donations of breast-milk substitutes in health facilities and marketed foods and beverages for infants under six months of age and young children up to 36 or 59 months of age [30,31]. This requires updating laws and monitoring systems and using effective penalties for violators of the Code and guidance on ending inappropriate marketing of food for infants and young children up to the age of 36 months [30] and control of labeling and provocative health claims of foods marketed for children up to 59 months of age [31].

Community-based interventions are crucial for empowering women to demand Baby-Friendly practices when giving birth in maternity centers. Raising awareness through the use of e-media such as social media and applications on mobile devices can promote optimal breastfeeding practices and protect parents and their children from misinformation propagated by inappropriate marketing. These interventions to promote and protect breastfeeding will assist countries to implement and scale-up with BFHI. Breastfeeding promotion can assist countries in overcoming the problems of malnutrition and the rising trends in NCDs and their effect on economic development in the region [32,33,34].

## Figures and Tables

**Table 1:** Status of the Baby-Friendly Hospital Initiative by number of designated hospitals in each country grouped by their nutrition stages and with dominant nutrition problems [17].

Group I: Advanced Nutrition Transition		Group II: Early Nutrition Transition		Group III: High Acute and Chronic Malnutrition		Group IV: Complex Emergency, Severe Child and Maternal Undernutrition	
Country	Designated Hospitals	Country	Designated Hospitals	Country	Designated Hospitals	Country	Designated Hospitals
Iran	376	Egypt	13	Djibouti	4	Afghanistan	18
Tunisia	141	Jordan	3	Iraq	15	Somalia	0
Kuwait	1	Lebanon	21	Pakistan	42	Syria	21
Bahrain	6	Morocco	17	Sudan	25	Yemen	0
Qatar	0	Palestine	0			Libya	0
UAE	15						
Oman	51						
Saudi Arabia	60						
Total (832)	650		57		86		39
Percent	78.12		6.85		10.34		4.69

Data from United Nations International Children’s Emergency Fund (UNICEF) report [17].

**Table 2:** Trends in percent designated Baby-Friendly hospitals over the period from 2004 to 2016 in the country groups under study [17].

Country by Group	2004 (%)	2007 (%)	2010 (%)	2015 (%)	2016 (%)
Group I: Advanced nutrition transition					
Iran	94	96	96	86	86
Tunisia	93	93	93	-	93
Kuwait	33	33	33	25	25
Bahrain	21	21	21	-	23
Qatar	0	50	50	50	50
United Arab Emirates	40	40	59	65	70
Oman	100	100	100	100	100
Saudi Arabia	1	2	7	24	24.1
Range	1–100	2–100	7–100	25–100	23–100
Mean percent	47.75	54.4	57.38	58.33	58.89
SD	±42.05	±37.50	±36.05	±31.32	±32.59
Group II: Early nutrition transition					
Egypt	3	3	3	0	5
Jordan	49	4	4	4	4
Lebanon	16	16	9	0	21
Morocco	17	38	38	5	5
Palestine	0	0	0	0	0
Range	0–49	0–38	0–38	0–5	0–21
Mean	17	12.2	10.8	1.8	7
SD	±19.43	±15.66	±15.55	±2.49	±8.09
Group III: Chronic malnutrition					
Djibouti	0	0	0	0	0
Iraq	55	49	59	-	21
Sudan	9	11	11	0	11
Pakistan	4	7	7	0	65
Range	0–55	7–49	7–59	0	11–65
Mean	17	16.75	19.25	0	24.25
SD	+25.59	+21.98	+26.89	0	+28.49
Group IV: Complex emergency situations					
Afghanistan	1	1	1	1	17.3
Libya	0	0	0	0	0
Somalia	0	0	0	0	2
Syrian Arab Republic	20	35	35	35	25
Yemen	0	0	0	0	0
Range	0–20	0–35	0–35	0–35	0–25
Mean	4.2	7.2	7.2	7.2	8.86
SD	±8.84	±15.55	±15.54	±15.5	±11.57

Data from United Nations International Children’s Emergency Fund (UNICEF) report [17].

**Table 3:** Breastfeeding indicators, nutritional status, and percent Baby-Friendly hospitals, over the period from 2004 to 2016, in countries with advanced nutrition transition (group I), early nutrition transition (group II), acute and chronic malnutrition (group III), and countries with complex emergency situations (group IV) (expressed as mean and standard deviation and correlation coefficients) [18].

Country by Group	Baby Friendly Hospitals 2004–2016 (%)		Timely Initiation of First Feed (%)	Exclusive Breastfeeding for 6 Months (%)	Continued Breastfeeding for 24 Months (%)	Overweight Prevalence (%)	Stunting Prevalence (%)	Wasting Prevalence (%)
**Group I (8)**								
Range	23–100		1–71.1	8.5–53.1	9–51	1.9–15	4.9–10.1	2.1–10.1
Mean	1378.33		21.37	19.87	25.33	8.29	9.55	9.65
SD	±32.88		±33.42	±15.90	±14.90	±4.57	±3.28	±3.98
**Group II (5)**								
Range	0–49		0–41.3	22.7–39.7	10.6–25	5.7–15.5	7.3–22.3	1.2–9.5
Mean	27		29.3	29.2	17.225	9.075	11.92	4.54
SD	±20.08		±9.39	±7.33	±6.34	±5.03	±6.637	±3.426
**Group III (4)**								
Range	0–65		18–68.7	1.3–55.4	18.4–56.1	3.1–10.1	22.6–45	7.433
Mean	22		28	20.48	21.931	4.569	33.875	17.3
SD	±29.59		±21.51	±23.29	±18.74	±3.93	±9.78	±11.32
**Group IV (5)**								
Range	0–35		0–53.6	5.3–43.3	24.9–58.6	4–20.15	21.0–42.1	6.5–16.3
Mean	13.6		43.8	26.1	38.9	11.6	34.66	10.54
SD	±19.66		±14.07	±21.25	±11.27	±6.88	±12.10	±4.1476
**Correlations with nutritional indices**								
**Children under five**	**Timely first suckle for all country groups**			**Exclusive breastfeeding for all country groups**		**Continued breastfeeding for 12 months & 24 months for all country groups**		
	First hour of life	*p*-value		(0–5 mo)	*p*-value	At 12 mo	At 24 mo	*p*-value
Stunting	*r* = 0.09	*p* > 0.05		*r* = 0.05	*p* > 0.05	*r* = 0.69 *	*r* = 0.908 **	*p* < 0.001 *p* < 0.01 *p* > 0.05
Wasting	*r* = 0.22	*p* > 0.05		*r* = 0.13	*p* > 0.05	*r* = 0.67 *	*r* = 0.87 **	
Overweight	*r* = 0.51 *	*p* < 0.05		*r* = 0.11	*p* > 0.05	*r* = 0.05	*r* = 0.36	
Obesity	*r* = 0.51 *	*p* < 0.05		*r* = 0.22	*p* > 0.05	*r* = 0.475 *	*r* = 0.33	

Country Groups include: Group I: 8 countries in advanced nutrition transition stage, Group II: 5 countries in early nutrition transition stage, Group III: 4 countries with significant undernutrition, Group IV: 5 countries in complex emergency.
